# The First Morpho-Molecular Study of *Shimizuomyces paradoxus* in Vietnam: Insights into Fungal Biodiversity in Bidoup National Park

**DOI:** 10.3390/life15040644

**Published:** 2025-04-14

**Authors:** Giang Van Nguyen, Binh Van Nguyen, Hue Hong Thieu, Hanh Ngoc Nguyen, Thuy Ai Huyen Le, Nguyen Binh Truong, Thuan Duc Lao

**Affiliations:** 1 Faculty of Biology, Dalat University, Lam Dong 670000, Vietnam; giangnv@dlu.edu.vn (G.V.N.); binhnv@dlu.edu.vn (B.V.N.); 2 Center of Life Science Research, Ho Chi Minh City Open University, Ho Chi Minh City 722700, Vietnam; hue.th@ou.edu.vn (H.H.T.); thuy.lha@ou.edu.vn (T.A.H.L.); 3 Faculty of Biotechnology, Ho Chi Minh City Open University, Ho Chi Minh City 722700, Vietnam

**Keywords:** *Shimizuomyces paradoxus*, entomopathogenic fungi, morphology analysis, phylogenetic analysis

## Abstract

A fungal specimen, designated as DL0035, was found to parasitize the seed of *Smilax* sp. in the area of Bidoup National Park, Nui Ba, in Lam Dong Province, Vietnam. The identification of the species was typically identified both by morphological and molecular phylogenetic analysis. Total genomic DNA was isolated by the method of Phenol/chloroform. The target gene *ITS* and *nrLSU* were amplified and sequenced by PCR and the Sanger method. Then, the sequence of the sample was subjected to molecular phylogenetic analysis to assist with species identification. The results of morphological analysis confirmed that the sample of DL0035 is *Shimizuomyces paradoxus* Kobayashi and Shimizu. Moreover, based on the phylogenetic analysis, the DL0035 was formed with the referent sequence of *Shimizuomyces paradoxus* with the high bootstrap value. Therefore, based on morphology and phylogenetic analysis, the sample of DL0035 was identified as *Shimizuomyces paradoxus*. Notably, this was the first record of *Shimizuomyces paradoxus* species in Vietnam.

## 1. Introduction

The family of Clavicipitaceae (Hypocreales, Ascomycota), historically, consisted of 43 genera in 2008. Recent advancements in research and taxonomy have greatly improved our understanding of this group. By the year of 2020, the number of genera within the family had increased to 50 [1,2]. This family encompasses a diverse species, including both saprotrophic and symbiotic fungi, which associate with insects and fungi or grasses, rushes, and sedges for genera such as *Cordyceps* spp., *Balansia* spp., *Epichloë* spp., and *Claviceps* spp. [3].

The genus *Shimizuomyces* was described by Kobayashi in Japan as part of the *Clavicipitaceae* family [4,5,6,7]. Furthermore, the genus *Shimizuomyces* shares the similarity of morphological characteristics with the *Cordyceps* s.l., 1981, Kobayashi [8]. The genus *Shimizuomyces* was once again confirmed to belong to the family *Clavicipitaceae* upon the re-evaluation of fungal species in the genus Cordyceps collected from Japan, New Guinea, Formosa, China, and the United States. The species of *Shimizuomyces paradoxus* Kobayashi is the type species of the genus Shimizuomyces, serving as its defining representative in taxonomic classification [4,5,6,7].

Sung et al. (2007) provided confirmation of the taxonomic position of the *Shimizuomyces* genus within the family of *Clavicipitaceae* through their research on the molecular phylogeny of *Cordyceps* fungus and related species [6]. Previous reports stated that only two species of the genus of *Shimizuomyces* have been described. They parasitize the seeds of plants. To be specific, *Shimizuomyces paradoxus* parasitizes the seeds of *Smilax sieboldin* (Family *Smilacaceae*) (MB#114378), and *Shimizuomyces kibianus* parasitizes the seeds of *Smilax china* (Family *Smilacaceae*) (MB#281560). According to the description by Kobayasi Y (1984) [9], there are distinct morphological differences between these two species. *Shimizuomyces paradoxus* is characterized by club-shaped and yellowish fruiting bodies, while *Shimizuomyces kibianus* is characterized by elongated, egg-shaped, and earthy-yellow fruiting bodies. In addition, the ascospores of *Shimizuomyces paradoxus* are 60–75 µm in length, whereas those of *Shimizuomyces kibianus* are shorter at 30–40 µm. Furthermore, spindle-shaped cells that are situated in the middle of the ascospores are mostly seen in *Shimizuomyces paradoxus* and are seldom seen in *Shimizuomyces kibianus* [9]. The discovery of *Shimizuomyces paradoxus* in the region of Anhui was declared by Chinese scientists in 2008 [10]. As of 2010, *Shimizuomyces paradoxus* had been found in Korea [7]. Slight morphological variations in synnemata, perithecia, and asci have been described in the samples collected in these countries. The sample taken in Korea has bigger synemata than the one taken in Japan, although the asci and perithecia have smaller sizes. The oblong, slightly projecting synnemata grow gregariously at the base of the host fruits (the seeds of Smilax sieboldin), with the clear distinction between the stem and the reproductive part located at the end of the synnemata. Asci are spindle-shaped, with several septa and a single, sizable cell in the center [7,10]. As of yet, the species of *Shimizuomyces paradoxus* has not been documented in Vietnam.

Bidoup–Nui Ba National Park, a part of Langbiang World Biosphere Reserve recognized by UNESCO in 2015 and an ASEAN Heritage Park (2019), has been recorded as a significant center of biodiversity, one of the four biodiversity centers of Vietnam. Within its diverse ecosystem, Bidoup–Nui Ba National Park harbors various species of entomopathogenic fungi, which play a critical role in regulating insect populations and maintaining ecological balance [11,12,13,14,15].

The sample of DL0035 was discovered parasitizing the seed of *Smilax* sp. in the area of Bidoup National Park, Nui Ba, in Lam Dong Province, Vietnam. Notably, the morphological and phylogenetic analysis indicated DL0035 is *Shimizuomyces paradoxus*. This is the first recorded species of the genus *Shimizuomyces* discovered in Vietnam.

## 2. Materials and Methods

### 2.1. Fungal Specimen Collection

The specimen, DL0035, used for this study was collected on 13 September 2016, from the forest litter of a broad-leaved forest within the area of K’long K’lanh, Langbiang Biosphere Reserve (coordinates: 12°2′19.0″ N, 108°26′04.7″ E, elevation of 1680 m). To preserve its integrity, the specimen was immediately and carefully wrapped in wax paper, securely stored in a collection bag, and subsequently transported to the laboratory for detailed examination and analysis.

### 2.2. Morphological Study

Morphological observations of specimens were systematically carried out and meticulously recorded according to the guidelines of Kobayasi and Sung et al. [6]. The macroscopic characteristics of the fresh fruit body were carefully observed. The key attributes observed include stipe, stroma, etc. The stroma, an integral component of the fruiting body, was carefully evaluated for its shape, size, color, and surface texture, as these features are critical for species identification and classification. Similarly, the stipe was examined for its length, diameter, color, and structural integrity, which contribute to the overall morphology and provide insight into the specimen’s developmental stage. The external features, such as the presence of mycelial coverings or other surface ornamentations, were also recorded in detail to ensure accurate characterization. The records of morphological features enabled the comparative studies.

### 2.3. DNA Extraction, PCR Amplification, and Target Sequencing

Total genomic DNA was isolated by the method of Phenol/Chloroform (pH = 8) within supplemented β-mecaptoethanol and CTAB [16]. A 1 g sample was incubated in a lysis buffer (2.0% SDS, Tris–HCl pH 8.0, 150 mM NaCl, 10 mM EDTA, 0.1 mg/mL Proteinase K) at 65 °C overnight. The supernatant was collected by centrifugation, and a volume of 700 μL of phenol/chloroform/isoamyl alcohol (25:24:1) was supplemented and centrifuged. The supernatant was collected and precipitated with absolute isopropanol. Finally, the isolated genomic DNA was stored in Tris–EDTA buffer at −20 °C for further studies.

The target genes of *ITS* and *nrLSU* were amplified by using the corresponding primers (Table 1). The final volume of PCR was done in a total of 15 μL with the thermal program: 1 cycle at 95 °C for 5 min, 40 cycles at 95 °C for 30 s, 55 °C for 30 s, 72 °C for 2 min, 1 cycle at 72 °C for 5 min; 5 μL aliquots of amplification product were electrophoresed on a 2.0% agarose gel and visualized in a UV transilluminator. The amplified product was sequenced at Nam Khoa Biotek Co., Ltd. (Ho Chi Minh City, Vietnam), following the molecular identification via phylogeny analysis.

### 2.4. Taxa and ITS, nrLSU Sequences Collection, DNA Proofreading and Phylogeny Analysis

The data set of *ITS* and *nrLSU* sequences were established by sequences downloaded from Genbank (NCBI) and based on the previous data. The sequences of *ITS* were noted with accession number and name of taxon. The amplified DNA sequences were proofread to remove ambiguous signals at both ends by different software, including Seaview 4.2.12 and Chromas Lite 2.1.1. Additionally, the best evolution model was predicted using jModelTest [19]. The phylogenetic tree was constructed based on maximum likelihood (ML), using Molecular Evolutionary Genetics Analysis (MEGA) version 11 [20].

## 3. Results

### 3.1. Taxonomy

*Shimizuomyces paradoxus* Kobayashi & Shimizu, 1981.

### 3.2. Distribution

**Figure 1:**
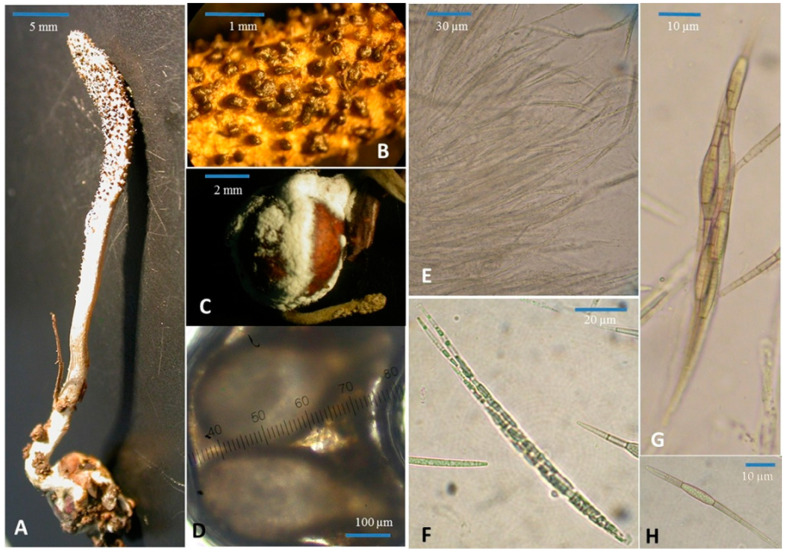
Morphological characteristics of DL0035; (**A**) fruiting body; (**B**) Fertile part surface; (**C**) mycelia on the seed of *Smilax* sp; (**D**) perithecia; (**E**,**F**) Asci; (**G**,**H**) Ascospore.

Shimizuomyces paradoxus is currently known from Japan, Korea [7,9,10], and Vietnam (current study) (Figure 1).

### 3.3. Typification

VIETNAM. Lam Dong Province, K’long K’lanh, Langbiang Biosphere Reserve. Coordinates: 12°02′19.0″ N, 108°26′04.7″ E; elevation of 1680 m; humidity: over 85%; temperature: day 20–22 °C, night: 14–16 °C; collected between 9–15 h of the day on 13 September 2016.

### 3.4. Morphology Observation

*Stomata*: 1, 2, or 3 stomata rise from the seeds of *Smilax sieboldin* and are covered by white mycelium. The stomata are 22–65 mm long, 1.5–3.0 mm in diameter, pale lemon-yellow, upright, and unbranched. *Fertile portion*: located at the head of the stomata, opalescent, 9–50 mm in size. *Stem part*: opalescent, 10–15 mm × 1.0–2.0 mm in size. Perithecia: dark brown, shape variations, distributed irregularly on the surface of the stomata, extended pear-shaped, 450–500 µm × 200–230 µm in size. Perithecian mouths are found in the middle of structures that resemble regular hexagons. *Asci*: cylindrical, 100–234 µm × 6.5–7.8 µm in size, thickened cap. *Ascospores*: filiform, multiseptate, consisting of a chain of 5–7 cells, 65–104 µm long. The largest cell is the middle one, 7.8 µm × 13–15.6 µm in diameter. It is typically tightly packed and inseparable, coiling within sporangia.

### 3.5. The Amplification of ITS and nrLSU

**Figure 2:**
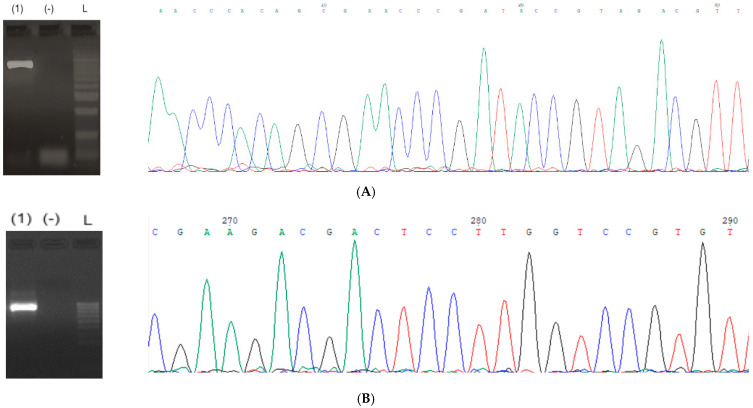
The electrophoresis for PCR product of *ITS*. (-) Negative control, L: ladder; and signal peaks of part of the sequence of (**A**) *ITS* and (**B**) *nrLSU*.

The target gene of *ITS* was successfully amplified by using corresponding primers, as detailed in Table 1. The results of the amplification, the bands of 1030-bp, corresponding to the amplified *ITS*, were observed on a 2.0% agarose gel (Figure 2A). Additionally, the appearance of clear and specific bands provided strong evidence for the successful amplification of the ITS region, demonstrating the specificity and efficiency of the primer design and amplification protocol. To confirm the accuracy, the PCR product was subjected to Sanger sequencing. The sequencing results for both strands of the *ITS* amplification product were high-quality data, as evidenced by chromatograms with significant, unique, and unambiguous peaks (Figure 2). This robust sequence clarity underscored the precision of the amplification process and confirmed the accuracy of the obtained genetic data of *ITS*.

### 3.6. Molecular Phylogeny Analysis

The reference sequence data set utilized for the phylogenetic analysis was obtained from Genbank (Genbank, NCBI) [21]. The dataset included thirty and nineteen referent sequences representing species from the *Cordyceps* genus and other genera within the family *Clavicipitaceae* for each of the target *ITS* and *nrLSU* genes, respectively. For the phylogeny tree analysis, two sequences of an outgroup, *Glomerella cingulate*, were incorporated into the data. (Table 2). The model of the general time reversible model with gamma distribution and invariable sites (GTR+G+I) and Tamura-Nei with gamma distribution (TN93+G) were identified as the optimal evolutionary model and applied to construct a phylogenetic tree using Maximum Likelihood (ML) methods within a replication of 1000.

**Figure 3:**
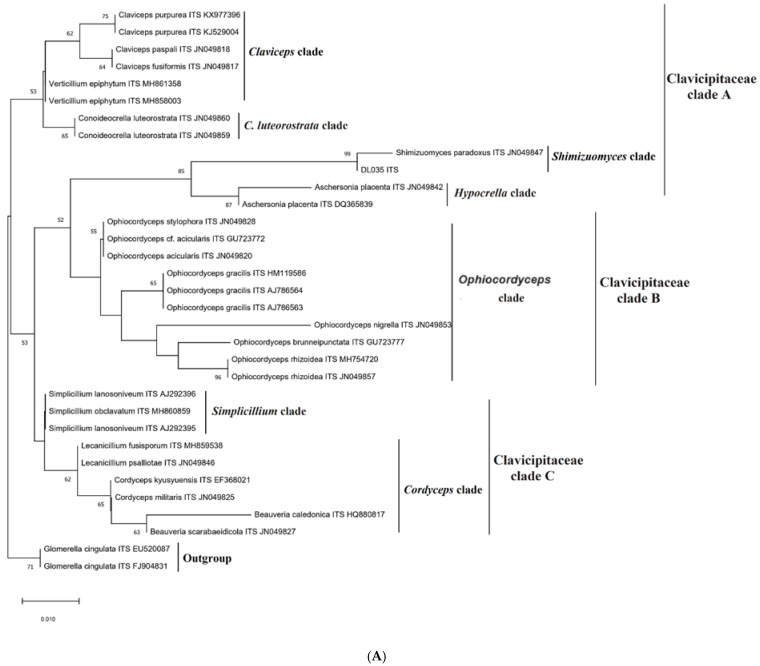

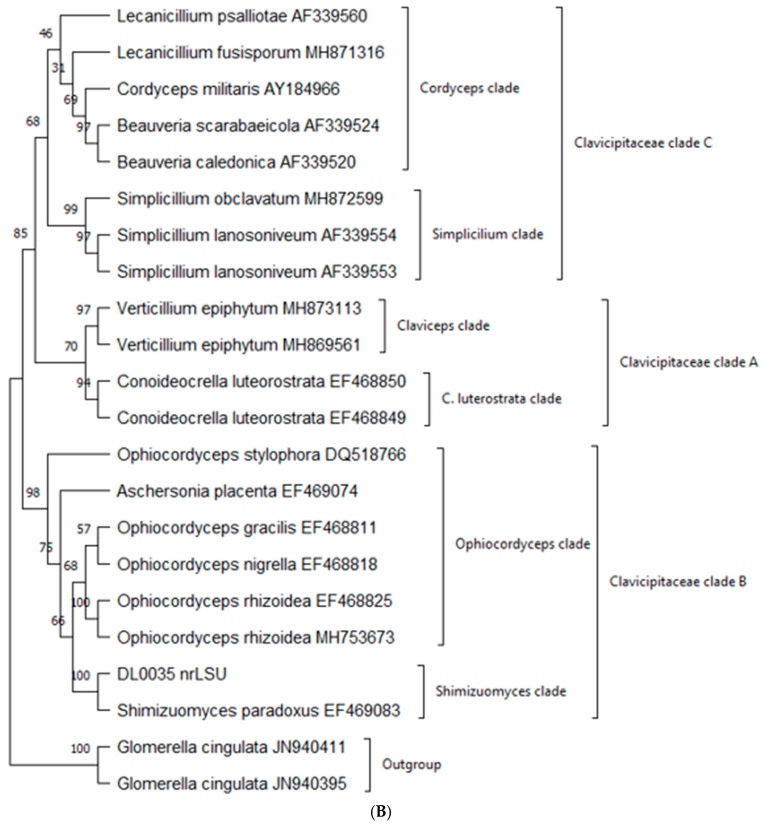
The phylogenetic relationship among DL0035 and its allies based on (**A**) *ITS* data and (**B**) *nrLSU* data. Bootstrap values (1000 replicates) are indicated above the nodes.

As a result, the topology provided a clear division of three main clades: Clavicipitaceae Clade A (*Claviceps*, *Metacordyceps*, *Balansia*, *Conoideocrella*, *Verticillium*), Clavicipitaceae Clade B (*Ophiocordyceps*), and Clavicipitaceae Clade C (*Simplicillium*, *Lecanicillium*), and are significantly separated from the clade of the outgroup (Figure 3). These three clades corresponded to the families of *Clavicipitaceae*, *Ophiocordycipitaceae*, and *Cordycipitaceae*.

Regarding the sample of DL0035, the DL0035 clustered with *Shimizuomyces paradoxus* (Accession number: JN049847, EF469083), with significant bootstrap, formed a separate monophyletic branch. Within this monophyletic branch, DL0035 and *Shimizuomyces paradoxus* (Accession number: JN049847, EF469083) clustered together closely, by observation of bootstrap values of 99 and 100 for *ITS* and *nrLSU*, respectively, suggesting a true phylogenetic relationship between these species. Additionally, the molecular phylogenetic analysis indicated strong and clear distinctions between DL0035 and other related species, underscoring its unique genetic and evolutionary position. These phylogentic analysis shed valuable insights into the taxonomic and evolutionary placement of DL0035 within the Clavicipitaceae family.

## 4. Discussion

The species of *Shimizuomyces paradoxus* is one of only two recorded species in the genus of *Shimizuomyces*. This species has been reported in various countries across the Asian region, including China, Japan, and Korea [7]. Thus, it demonstrated that the distribution of *Shimizuomyces paradoxus* is predominantly associated with the habitat of temperate and subtropical climates. However, it is noteworthy that, prior to the current study, there have been no documents of the presence of *Shimizuomyces paradoxus* in Vietnam.

Bidoup-Nui Ba National Park, the core area of the UNESCO-recognized Lang Biang Biosphere Reserve, is one of four centers of biodiversity in Vietnam. The Lang Biang Biosphere Reserve has been renowned for its rich and diverse ecosystem, including a variety of entomopathogenic fungi [12,14,22]. These fungi, which parasitize and infect insects, play a crucial ecological role in regulating insect populations and maintaining ecosystem balance. During a field expedition to Bidoup–Nui Ba National Park, Lam Dong province, Vietnam, the entomopathogenic fungi of DL0035 were collected on the litter of the broad-leaved forest in the area of K’long K’lanh, Langbiang Biosphere Reserve (12°2′19.0″ N, 108°26′04.7″ E, elevation 1680 m) on 13 September 2016. The collection of the sample of *Shimizuomyces paradoxus* from Bidoup–Nui Ba National Park, along with its subsequent morphological and molecular identification, represented the first report of this species in Vietnam. This finding not only widely provided the known geographical range of *Shimizuomyces paradoxus* but also pointed out the ecological significance of Bidoup–Nui Ba National Park as a habitat for diverse and previously unrecorded fungal species.

Table 3 presents a comparative analysis of the morphological characteristics of *Shimizuomyces paradoxus* samples collected from Vietnam (DL0035, current study) and previously recorded specimens from China, Korea, and Japan. The comparison encompassed morphological features, including fruiting body size, stem dimensions, fertile regions, perithecia, asci, and ascospores. In general, the fruit body size of DL0035 (22–65 mm) was larger than that of samples from China (16.7–24 mm) and Japan (15–40 mm). Regarding feature of the stem and fertile, those characteristics in DL0035 were distinct, with the size of them being significantly larger than those reported in other countries. Similarly, the size of perithecia in the current sample (450–500 µm × 200–230 µm) was slightly larger than in other samples. The Vietnamese asci (100–234 µm × 6.5–7.8 µm) were noticeably longer than those from other regions, emphasizing a unique morphological feature. Specifically, the ascospores of DL0035 exhibited a chain-like structure with a distinctly enlarged spore in the center, which is reportedly a highly distinctive characteristic of *Shimizuomyces paradoxus* [7,8,10]. This morphological analysis highlighted the distinct features of the Vietnamese *Shimizuomyces paradoxus* specimen, contributing valuable insights into the diversity and adaptation of the species across its geographical range.

The region of the *internal transcribed spacer* (*ITS*) served as a standard marker and the first marker of diagnosis for DNA barcoding, particularly in molecular genealogical analysis of many organisms, including fungi, plants, and some protists [23,24,25]. It can be explained that the region of target genes shows significant sequence variation among different species, making it ideal for species-level identification, as well as a broader range of fungi [26,27]. Regarding molecular genealogical analysis, the phylogenetic tree was built based on the database of *ITS* and *nrLSU* genes with a total sequence of 30, 19 sequences in the *Clavicipitaceae* family, and an outgroup sequence of 2 sequences. The phylogenetic tree results show the high support with the bootstrap value reaching 100 in the monophyletic group, which clustered from DL0035 and *Shimizuomyces paradoxus EFCC* 6279 (JN049847, EF469083), belonging to the *Shimizuomyces* genus, Clavicipitaceae A. Additionally, this monophyletic group is also completely separate from other species of the Clavicipitaceae A group (Figure 3). These results are completely similar to the study of Sung et al. (2007) [6]. Thus, based on phylogeny analysis, this result completely supports the morphology-based identification: the sample of DL0035 is the species of *Shimizuomyces paradoxus* Kobayashi & Shimizu.

## 5. Conclusions

The sample of DL0035, which was found to parasitize the seed of *Smilax* sp. in the area of Bidoup National Park, Nui Ba, in Lam Dong Province, Vietnam, is morphologically and phylogenetically identified as *Shimizuomyces paradoxus* Kobayashi & Shimizu, Shimizuomyces genus, *Clavicipitaceae*. In addition, the sample of DL0035 represents the first documented instance of *Shimizuomyces paradoxus* in Vietnam.

## Figures and Tables

**Table 1:** The sequences of primers used in current study.

Target Gene	Primer	Sequence (5′–3′)
*ITS* [17]	ITS1F (F)	TCCGTAGGTGAACCTGCGG
	ITS4 (R)	TCCTCCGCTTATTGATATGC
*nrLSU* [18]	LROR (F)	GTACCCGCTGAACTTAAGC
	LR5 (R)	ATCCTGAGGGA AACTTC

Note: F: Forward primer, R: reversed primer.

**Table 2:** Representative taxa information and GenBank accession numbers for sequences used in current study.

STT	Taxon	Genus	Strain	*ITS*	*nrLSU*
1	*Ophiocordyceps* cf. *acicularis*	*Ophiocordyceps*	NHJ12622	GU723772	n/a
2	*Ophiocordyceps acicularis*	*Ophiocordyceps*	OSC128580	JN049820	n/a
3	*Claviceps fusiformis*	*Claviceps*	ATCC 26019	JN049817	n/a
4	*Claviceps paspali*	*Claviceps*	ATCC 13892	JN049818	n/a
5	*Verticillium epiphytum*	*Verticillium*	CBS 154.61	MH858003	MH869561
6	*Verticillium epiphytum*	*Verticillium*	CBS 384.81	MH861358	MH873113
7	*Claviceps purpurea*	*Claviceps*	SS-F26	KJ529004	n/a
8	*Claviceps purpurea*	*Claviceps*	PA1	KX977396	n/a
9	*Conoideocrella luteorostrata*	*Conoideocrella*	NHJ 12516	JN049860	EF468849
10	*Conoideocrella luteorostrata*	*Conoideocrella*	NHJ 11343	JN049859	EF468850
11	*Shimizuomyces paradoxus*	*Shimizuomyces*	EFCC 6279	JN049847	EF469083
12	*Simplicillium obclavatum*	*Simplicillium*	CBS:311.74	MH860859	MH872599
13	*Ophiocordyceps rhizoidea*	*Ophiocordyceps*	NHJ 12522	JN049857	EF468825
14	*Ophiocordyceps rhizoidea*	*Ophiocordyceps*	BCC 48879	MH754720	MH753673
15	*Ophiocordyceps nigrella*	*Ophiocordyceps*	EFCC 9247	JN049853	EF468818
16	*Ophiocordyceps gracilis*	*Ophiocordyceps*	2684.S	AJ786563	n/a
17	*Ophiocordyceps gracilis*	*Ophiocordyceps*	2685.4	AJ786564	n/a
18	*Ophiocordyceps gracilis*	*Ophiocordyceps*	n/a	HM119586	EF468811
19	*Ophiocordyceps stylophora*	*Ophiocordyceps*	OSC 111000	JN049828	DQ518766
20	*Beauveria scarabaeicola*	*Beauveria*	ARSEF 5689	JN049827	AF339524
21	*Ophiocordyceps brunneipunctata*	*Ophiocordyceps*	NHJ1491	GU723777	n/a
22	*Beauveria caledonica*	*Beauveria*	ARSEF 2567	HQ880817	AF339520
23	*Aschersonia placenta*	*Hypocrella*	BCC 7869	JN049842	EF469074
24	*Aschersonia placenta*	*Hypocrella*	NHJ6225	DQ365839	n/a
25	*Simplicillium lanosoniveum*	*Simplicillium*	CBS 704.86	AJ292396	AF339553
26	*Simplicillium lanosoniveum*	*Simplicillium*	IMI 317442	AJ292395	AF339554
27	*Lecanicillium fusisporum*	*Lecanicillium*	CBS:164.70	MH859538	MH871316
28	*Lecanicillium psalliotae*	*Lecanicillium*	CBS 532.81	JN049846	AF339560
29	*Cordyceps kyusyuensis*	*Cordyceps*	HMAS 78115	EF368021	n/a
30	*Cordyceps militaris*	*Cordyceps*	OSC 93623	JN049825	AY184966
31 *	*Glomerella cingulate*	*Colletotrichum*	NW677b	EU520087	JN940395
32 *	*Glomerella cingulate*	*Colletotrichum*	Gr212	FJ904831	JN940411

Note: * outgroup, n/a: not-provided.

**Table 3:** Comparison of morphological characteristics between DL0035 and *Shimizuomyces paradoxus* collected in China, Korea, and Japan.

The Morphological Characteristics		*Shimizuomyces paradoxus*			
		DL0035 (Current Study, Vietnam)	China [10]	Korea [7]	Japan [9]
Fruiting bodies	Size	22–65 mm	16.7–24 mm	NA	15–40 mm
	Stem	10–15 mm × 1.0–2.0 mm	NA	4–38 mm	10–30 mm × 0.5–1.2 mm
	Fertile	9.0–50 mm × 1.5–3.0 mm	2.2–5.9 mm × 1.2–1.5 mm	3–17 mm	5.0–15 mm × 1.0–2.0 mm
Perithecia		450–500 µm × 200–230 µm	320–390 µm × 180–270 µm	300–500 µm × 150–300 µm	350–400 µm × 200–250 µm
Asci		100–234 µm × 6.5–7.8 µm	90–150 µm × 6.5–7 µm	100–130 µm	90–130 µm
Ascospore		65–104 µm Cylindrical, a chain of 5–7 cells. The largest cell: middle, 7.8 µm × 13–15.6 µm in diameter.	55–87.5 µm × 3–3.5 µm Cylindrical, a chain of 3–14 cells.	60–70 µm Cylindrical, no information: amount cells.	60–75 µm × 2–2.5 µm Cylindrical, a chain of 3–7 cells.

## Data Availability

The original contributions presented in this study are included in the article. Further inquiries can be directed to the corresponding authors.
